# Green Synthesis of Castor Oil-Modified Waterborne Polyurethanes via a Solvent-Free Approach

**DOI:** 10.3390/polym18121449

**Published:** 2026-06-10

**Authors:** Angus Shiue, Kai-Yen Chin, Yu-Han Liu, Shu-Mei Chang, Graham Leggett

**Affiliations:** 1 Department of Molecular Science and Engineering, National Taipei University of Technology, Taipei 106, Taiwan; angusshiue@gmail.com (A.S.); f104gstyle@gmail.com (K.-Y.C.); xuan518023@gmail.com (Y.-H.L.); 2 Process Insights—Tiger Optics, LLC, 275 Gibraltar Rd., Horsham, PA 19044, USA; graham.leggett@licor.com

**Keywords:** castor oil, waterborne polyurethane, nonionic, solvent-free

## Abstract

The conventional production of waterborne polyurethane (WPU) typically relies on organic solvents to regulate viscosity; additionally, traditional ionic WPU systems still utilize volatile neutralizers, raising environmental and health concerns. To overcome these limitations and reduce dependence on petrochemical resources, this study presents a solvent-free approach for WPU synthesis using isophorone diisocyanate (IPDI), polytetrahydrofuran (PTMG), and the nonionic PEG derivative Ymer^TM^ A-130. In addition, castor oil (CO), a renewable and hydroxyl-rich bio-based material, was incorporated as a partial substitute for PTMG to improve both sustainability and material performance. The effects of varying substitution ratios of castor oil on the physical properties of the resulting dispersions, dried films, and coatings were initially investigated. The results indicate that increasing the castor oil content from 0 wt% to 11.8 wt% led to an enhancement in tensile strength, rising from 1.45 MPa to 2.40 MPa. Concurrently, the temperature at 5% weight loss (Td_5%_) shifted upward from 263.84 °C to 285.36 °C, indicating a favorable trend in thermal stability. Furthermore, the preliminary solvent resistance, surface wetting characteristics, and environmental durability of the prepared coatings were evaluated and discussed.

## 1. Introduction

Waterborne polyurethanes (WPUs) drastically lower volatile organic compound (VOC) emissions by employing water as a dispersion medium [1,2]. Despite this eco-friendly advantage, conventional WPU synthesis remains dependent on non-renewable, petrochemical-derived raw materials [3]. To mitigate fossil resource depletion and environmental footprints, the development of biomass-derived alternatives has become a key priority in polymer research.

Consequently, bio-based polyols sourced from sustainable materials—such as starch, lignin [4,5,6], and vegetable oils [7,8,9]—have gained prominence due to their low toxicity and cost-effectiveness [10,11,12]. Castor oil (CO) stands out as a highly effective renewable monomer. When formulated alongside polytetrahydrofuran (PTMG) and isophorone diisocyanate (IPDI), it yields high-performance bio-based WPUs [13]. Mechanistically, the multifunctional hydroxyl groups of CO establish a robust crosslinked network [14], complemented by the flexible soft segments of PTMG [15] and the weather-resistant, aliphatic hard segments of IPDI. This structural synergy ultimately optimizes the mechanical strength, hydrophobicity, and thermal stability of the resulting WPU films.

Due to increasingly stringent environmental policies and regulations, the polyurethane industry has gradually shifted from solvent-based polyurethane systems to WPU coatings. However, during the synthesis of WPU—in both academic research and industrial production—a small amount of organic solvents, such as acetone and N-methyl-2-pyrrolidone (NMP), is often introduced. These solvents are used to reduce the viscosity of the prepolymer, thereby facilitating its emulsification in water to form a stable polyurethane dispersion [16,17].

Although the amount of solvent used in this process is significantly lower than that in conventional solvent-based polyurethane systems, concerns regarding solvent volatilization, environmental impact, and occupational safety persist. In addition, the post-synthesis removal of these solvents via vacuum concentration increases both energy consumption and operational costs. To address these issues, solvent-free preparation methods have been developed, in which no organic solvents are introduced during the synthesis process [18].

However, many reports still focus on using ionic agents to provide the hydrophilicity necessary to ensure the smooth dispersion of the prepolymer [19,20,21]. Particularly in reports involving renewable sources that utilize CO as a polyol, 2,2-bis (hydroxymethyl) propionic acid (DMPA) is generally used [22,23,24]. Yet, DMPA requires a toxic and volatile neutralizer, triethylamine (TEA), to neutralize the carboxylic acid groups into carboxylates to enhance the hydrophilicity of the WPU system. This dependency makes it difficult for such systems to comply with regulations in regions enforcing strict indoor air quality and environmental laws.

This study seeks an environmentally friendly approach to synthesize bio-based WPUs, enabling stable emulsification without organic solvents while minimizing the use of volatile neutralizers. To date, PEG derivatives (such as Ymer^TM^ N-120) have been reported as nonionic chain extenders in WPUs capable of yielding low-viscosity dispersions [25]. Nevertheless, their systematic utilization in the synthesis of solvent-free WPUs, where CO serves as a partial polyol substitute, remains unreported. In this research, a series of WPUs were designed based on IPDI and PTMG, utilizing CO as a partial substitute for petroleum-based polyols in combination with a nonionic PEG derivative (Ymer^TM^ A-130, supplied by Jollity Enterprise Co., Ltd., Taoyuan, Taiwan). This work provides an initial exploration into the effects of CO incorporation at different weight ratios on the properties of the WPU dispersions, as well as the fundamental mechanical performance, thermal stability of the dried films, and overall weather resistance as coatings.

## 2. Materials and Methods

### 2.1. Synthesis of Castor Oil-Modified WPUs (CWPUs)

This study does not employ organic solvents, such as acetone, ethanol, or NMP, for controlling the viscosity of the prepolymer system or dissolving monomers during the reaction. Instead, a solvent-free synthesis was carried out in a moisture-sealed three-necked flask equipped with a mechanical stirrer and a condenser.

**Figure 1:**
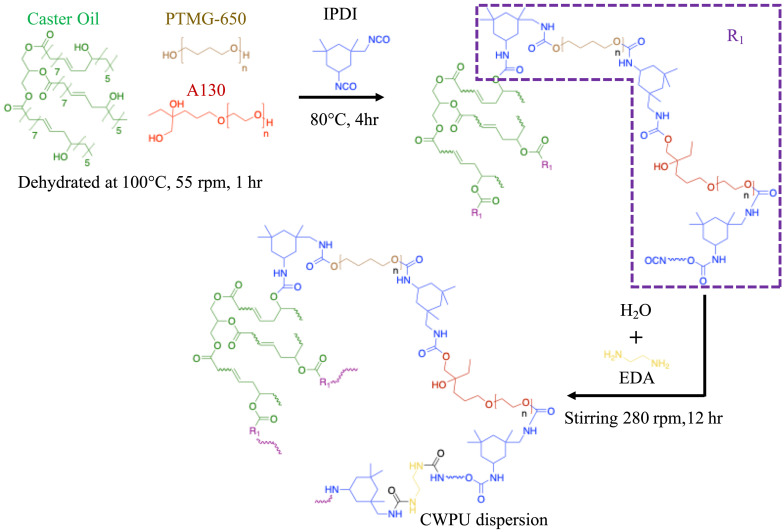
Reaction process scheme of CWPU.

The synthesis process of castor oil-modified waterborne polyurethanes (CWPUs) is illustrated in Figure 1, and the corresponding molar ratios of each reagent are summarized in Table A1. Polytetrahydrofuran (PTMG, Mn = 650 g/mol, Sigma-Aldrich, St. Louis, MI, USA), castor oil (CO, First Chemical Co., Ltd., Taipei, Taiwan), and PEG derivatives (Ymer^TM^ A-130, supplied by Jollity Enterprise Co., Ltd., Taoyuan, Taiwan) were vacuum-dehydrated at 100 °C (55 rpm) for 1 h, followed by the introduction of a nitrogen atmosphere. The system was then cooled to 80 °C, after which isophorone diisocyanate (IPDI, Sigma-Aldrich, St. Louis, MI, USA) and dibutyltin dilaurate (DBTDL, Sigma-Aldrich, St. Louis, MI, USA) were added, and the prepolymerization proceeded for 4 h.

Once the temperature decreased to 55 °C, deionized water was introduced under high-speed stirring (500 rpm) for 5 min to disperse the prepolymer. Chain extension was subsequently carried out by the gradual addition of diluted 1,2-diaminoethane (EDA, Sigma-Aldrich, St. Louis, MI, USA) in deionized water, followed by stirring at 280 rpm for 12 h. The resulting CWPU dispersion was then cast into a mold, air-dried at room temperature for 3 days, and further dried at 40 °C for 12 h to obtain the final CWPU film. The sample codes were designated based on the weight percentage of castor oil relative to the total monomer content, ranging from CWPU0 to CWPU11.8.

### 2.2. Parallel Coating of Dispersion

Glass substrates were sequentially cleaned with a mild detergent, acetone (99.5%, Echo Chemical Co., Ltd., Toufen, Taiwan), and ethanol (95%, Echo Chemical Co., Ltd., Toufen, Taiwan), followed by thorough drying. The cleaned glass substrates were positioned on a motorized parallel coating machine. The WPU dispersion was then cast using a 25 μm doctor blade at a constant coating speed of 20 cm min^−1^ to ensure a uniform distribution. Finally, the coated samples were dried in an oven at 50 °C for 12 h to remove residual water and yield the final CWPU films.

### 2.3. Characterizations

FTIR spectra were obtained using a PerkinElmer spectrum (Waltham, MA, USA). The particle size of the CWPU dispersion was measured using DLS (Nano Brook 90 Plus, Brookhaven, GA, USA) measurement. The viscosity of the CWPU dispersion was determined with a DV-E viscometer (Brookfield, WI, USA), using an LV-4 spindle for 60 s at 125 s^−1^ and 25 °C. The mass loss of all samples was determined by TGA (TG 209 F3, Netzsch, Selb, Bavaria, Germany), with a heating rate of 10 °C/min from 30 to 700 °C in N_2_. The static water contact angle (WCA) of the coatings was measured by a Phoenix 150 contact-angle meter through a 5 μL distilled water droplet at five different positions on each sample. Mechanical testing was performed using a universal testing machine (A2 TYPE, HUNG TA, Taichung, Taiwan) at a crosshead speed of 500 mm/min. Specimens were die-cut into a Type V dumbbell shape (0.9 ± 0.1 mm thickness), in accordance with ASTM D638 [26].

#### 2.3.1. Isocyanate Content Titration

The isocyanate (–NCO) group content of the prepolymer was determined via back-titration according to ASTM D2572 [27]. Approximately 1 g of sample was dissolved in 25 mL of toluene, followed by the addition of 25 mL of 0.1 N di-n-butylamine. After 30 min of reaction, 100 mL of isopropanol and bromophenol blue indicator were added. The solution was titrated with 0.1 N HCl to a yellow endpoint (V_1_). A blank titration (V_0_) was performed under identical conditions. The actual NCO% was calculated from these results and compared to the theoretical value.

(1) $$Theoretical\;NCO\;(\%)=\frac{\left(Equivalent\;weight\;of\;NCO-Equivalent\;weight\;of\;OH\right)\times 0.042}{Total\;weight\;of\;the\;reactants}$$

(2) $$Titrated\;NCO\;(\%)=\frac{\left(V_{0}-V_{1}\right)\times N\times 0.042}{W}$$

where V_0_ and V_1_ are the volumes of HCl (mL) consumed by the blank and the sample, respectively; N is the normality of the HCl solution (mol/L); W is the sample weight (g); and 0.042 represents the milliequivalent weight of the NCO groups.

#### 2.3.2. Calculation of Crosslinking Density

The crosslinking density (*n*) of the WPU films was determined using the Flory–Rehner equation based on solvent swelling [28]. Samples (5 mm × 5 mm) were weighed, measured for thickness, and immersed in toluene for 24 h to reach swelling equilibrium. The swollen samples were then removed, wiped dry, and re-measured. The crosslinking density was calculated using the following parameters:

(3) $$-[\ln\left(1-v_{2}\right)+v_{2}+\chi v_{2}^{2}]=v_{1}\times n[v_{2}-\frac{v_{2}}{2}]$$

The crosslinking density was calculated using the following parameters: Molar volume of toluene (v_1_): 106.3 cm^3^/mol; Interaction parameter (χ): 0.225 (derived from solubility parameters δ_PU_ = 20.5 and δ_tol_ = 18.2 MPa^1/2^ at 303 K); Absolute temperature (T); and Ideal gas constant (R): 8.314 cm^3^·MPa/K·mol. The polymer volume fraction after swelling (v_2_) was determined by the ratio of the original volume to the equilibrium swollen volume.

(4) $$\chi =\frac{V_{1}\times {\left(\delta_{2}-\delta_{1}\right)}^{2}}{RT}$$

#### 2.3.3. Chemical Resistance

Chemical resistance was evaluated according to ASTM D543-21 [29] by immersing WPU films in tetrahydrofuran (THF), methyl ethyl ketone (MEK) and toluene for 24 h. The initial weight of the film (W_0_) was recorded before immersion. After 24 h, the samples were removed, wiped dry to remove surface solvent, and weighed again to obtain the final weight (W_1_). The relative weight ratio (%) was calculated as follows:

(5) $$Relative\;weight\;ratio\;(\%)=\frac{W_{1}}{W_{0}}\times 100\%$$

#### 2.3.4. Water Absorption

Water absorption was evaluated following the ASTM D570-22 standard [30]. Film specimens (1 cm × 1 cm) were weighed (W_0_) and fully immersed in distilled water at room temperature. After 24 h or 48 h, the samples were removed, blotted dry to eliminate surface moisture, and immediately reweighed (W_1_). The water absorption (%) was calculated as follows:

(6) $$Water\;absorption\;rate\;(\%)=\frac{W_{1}-W_{0}}{W_{0}}\times 100\%$$

## 3. Results and Discussion

### 3.1. Monitoring of the Polyurethane Synthesis

**Figure 2:**
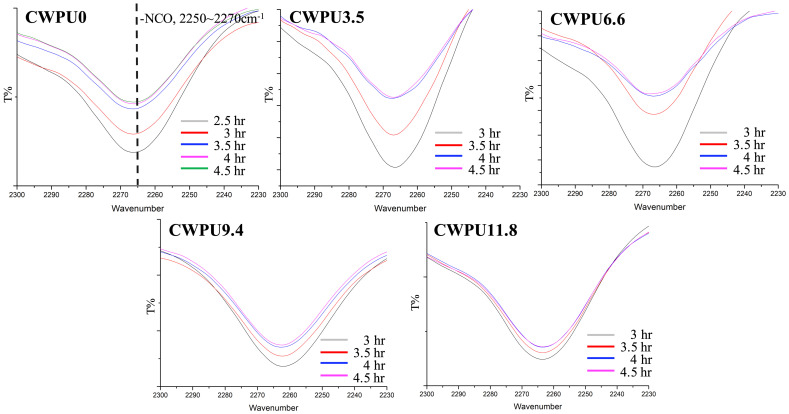
Variation of –NCO content during CWPU prepolymerization over time.

The prepolymerization progress was monitored via FT-IR spectroscopy and –NCO titration (ASTM D2572). As shown in Figure 2, FT-IR was used to track the characteristic isocyanate absorption peak at 2250–2270 cm^−1^. This peak intensity decreased rapidly as –NCO groups reacted with the hydroxyl (–OH) groups of PTMG and castor oil, eventually stabilizing after 4 h. This plateau indicated that the prepolymerization stage had reached completion, thereby establishing 4 h as the optimal reaction time for the synthesis process. To further confirm the extent of reaction, back-titration was conducted by reacting the prepolymer with an excess of di-n-butylamine in toluene, followed by titration of the unreacted amine with a standard HCl solution. The reaction was considered complete when the experimentally determined NCO% was equal to or lower than the theoretical value (as summarized in Table A2, Table A3, Table A4, Table A5 and Table A6), indicating the complete consumption of hydroxyl groups from the polyols. A representative calculation for the theoretical NCO% remaining after complete –OH reaction is detailed below, using CWPU3.5 as an example.

### 3.2. Flow Behavior and Storage Stability of CWPU Dispersions

#### 3.2.1. Particle Size and Viscosity Analysis

The particle size and distribution of the CWPU dispersions were determined by DLS, while viscosity measurements were conducted to evaluate their flow behavior and colloidal stability—both critical parameters for industrial processing. Although dispersion properties are influenced by the post-chain extension process (reaction with water and EDA) and hydrophilic group ratios, the CO content served as the primary variable in this study.

**Figure 3:**
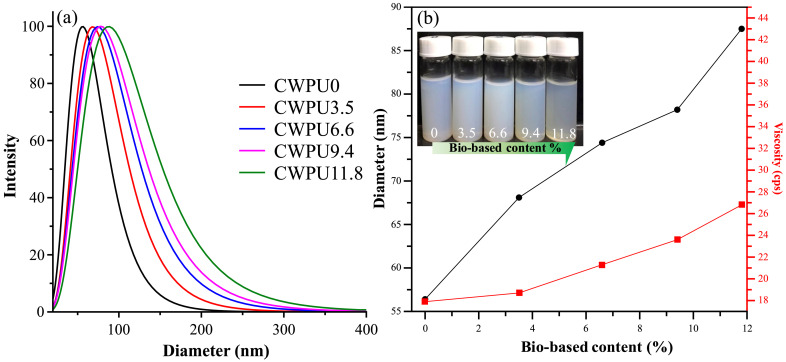
CWPU dispersion properties: (**a**) particle size distribution; (**b**) particle size and viscosity trends.

As shown in Figure 3a, all samples (CWPU0 to CWPU11.8) displayed a translucent bluish appearance, characteristic of the Tyndall effect in stable colloidal dispersions. As presented in Figure 3b and summarized in Table A7, both the average particle size and the breadth of the distribution increased gradually with increasing CO content. This trend suggests that the incorporation of the hydrophobic CO structure significantly influences the micellar formation and packing density of the polyurethane chains during the dispersion phase.

Larger particle sizes can be attributed to the fatty acid side chains of CO, which enhance the WPU backbone’s hydrophobicity and strengthen intermolecular attractions. Moreover, the trifunctional architecture of CO promotes the formation of a crosslinked network, further increasing particle dimensions [31]. As the CO content increases, the extent of this network structure also increases, resulting in greater molecular chain entanglement. Consequently, the dispersion and emulsification of the polyurethane prepolymer in water become more difficult. As a result, both the particle size and viscosity of the dispersion gradually increase [19].

#### 3.2.2. Storage Stability

CWPU dispersions may experience chain scission or particle aggregation over time due to environmental exposure and continuous Brownian motion. To evaluate long-term stability, the samples were stored in a cool, dark environment at room temperature and monitored every 10 days for signs of sedimentation, phase separation, or solidification.

As summarized in Table 1, all dispersions—regardless of CO content—maintained excellent fluidity and homogeneity throughout the 90-day observation period. No signs of stratification or precipitation were observed, indicating that these WPU systems possess a shelf life of at least 90 days under standard storage conditions. This demonstrates that the incorporation of CO does not compromise the colloidal stability of the dispersions.

### 3.3. Properties of CWPU Films

#### 3.3.1. FT-IR Analysis of CWPU Films

**Figure 4:**
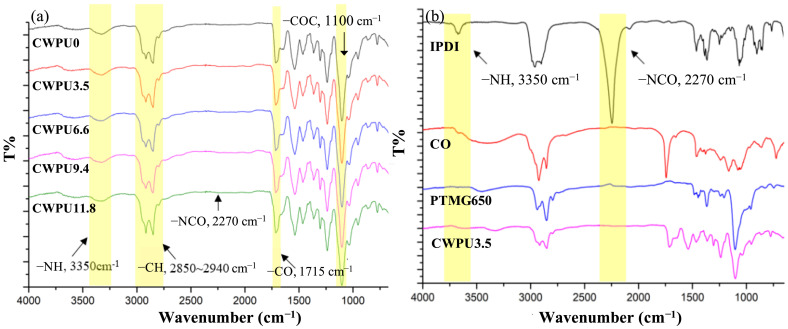
FT−IR spectra of (**a**) CWPU films with varying castor oil content and (**b**) comparison between the films and starting raw materials.

For subsequent characterization, CWPU films were prepared by casting and drying the dispersions. As shown in Figure 4a, FT-IR spectra confirmed the successful synthesis of the expected polymer architecture. Characteristic vibrations included N–H stretching/bending (3350, 1540 cm^−1^), asymmetric and symmetric –CH_2_ stretching (2940, 2850 cm^−1^), and C–O–C stretching from the PTMG soft segments (1100 cm^−1^). The complete absence of the 2270 cm^−1^ peak (Figure 4b) indicates total consumption of isocyanate (–N=C=O) groups.

#### 3.3.2. Crosslinking Density

The results of equilibrium swelling in toluene are summarized in Table 2, and the crosslinking density (*n*) is presented as a reference determined according to Section 2.3.2. First, it can be observed that the sample structure of CWPU0 (without CO) lost its integrity and partially dissolved after 24 h in toluene, indicating that the control sample lacks a crosslinked polymer network. With the incorporation of CO into the system, samples CWPU3.5 to CWPU11.8 exhibited stable swelling behavior, which serves as an indication of crosslinking within the material. The calculated density increased progressively from CWPU3.5 to CWPU11.8 (from 3.77 × 10^−2^ to 4.38 × 10^−2^ mol/cm^3^). This trend is attributed to the trifunctional triglyceride structure of CO, whereby a 3D urethane network is formed within the polymer matrix.

#### 3.3.3. Mechanical Properties

**Figure 5:**
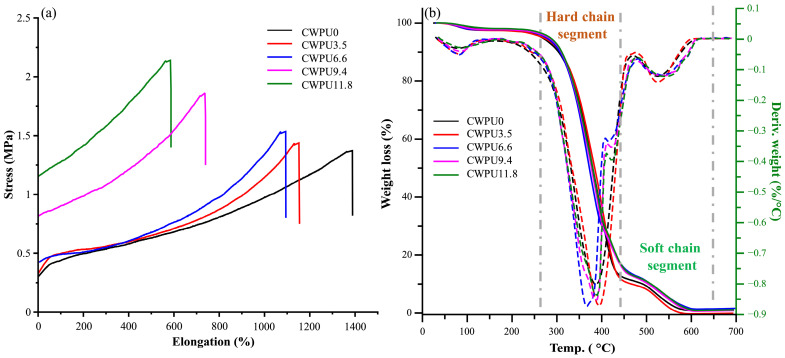
(**a**) Stress–strain curves and (**b**) TGA/DTG profiles of the CWPU films with varying castor oil content.

The stress–strain behavior of the CWPU films is presented in Figure 5a. With increasing CO content, the tensile strength rose from 1.45 MPa to 2.40 MPa, while the elongation at break decreased markedly from 1382.8% to 570.37%. Overall, the films transitioned toward higher stress and lower strain capacity.

This behavior can be attributed to the formation of a 3D cross-linked network and enhanced interchain hydrogen bonding within the hard segments. These structures restrict molecular chain mobility and inhibit chain slippage during deformation [24], thereby enhancing tensile strength at the expense of flexibility and elongation at break [32].

#### 3.3.4. Thermal Stability

The thermal stability of the CWPU films was evaluated via TGA under a N_2_ atmosphere, with the resulting thermograms presented in Figure 5b and Table 3. The decomposition process generally occurs in two stages: the first (260 to 430 °C) is associated with the degradation of the hard segments, while the second (430 to 600 °C) corresponds to the decomposition of the soft segments. However, a similar Td_max_ (The temperature of maximum thermal degradation) around 390 °C was observed for all samples, which is related to the dissociation of the urethane group [33]. Regarding the residual mass of all samples at 700 °C, the char residue remained below 2%.

As the CO content increased, the temperatures for the 5% (Td_5%_) and 10% (Td_10%_) weight losses rose from 263.84 °C and 303.84 °C to 285.36 °C and 310.36 °C, respectively. This enhancement is attributed to the incorporation of castor oil, which promotes a more robust crosslinked network. This dense structure restricts molecular chain mobility and acts as a barrier to thermal energy, thereby delaying the onset of degradation and significantly improving the thermal stability of the films [24].

#### 3.3.5. Chemical Resistance, Water Absorption, and Surface Wetting Properties

**Figure 6:**
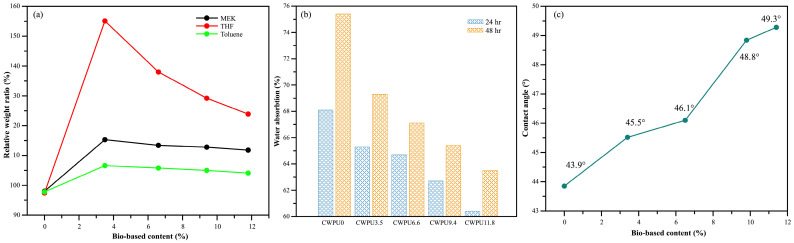
CWPU film properties as a function of castor oil content: (**a**) relative weight ratio after 24 h immersion, (**b**) water absorption, and (**c**) static contact angles.

The chemical and water resistance of the CWPU films were evaluated as described in Section 2.3.3 and Section 2.3.4, with results presented in Figure 6a,b. To quantify the chemical resistance of the CWPU films, the relative weight ratio after immersion was calculated using Equation (5), where a value of 100% signifies zero net mass change, while values exceeding 100% reflect weight gain from solvent uptake and swelling. As shown in Figure 6a, the control sample (CWPU0) exhibited a ~2% weight loss in MEK, THF, and toluene solvents, which can be reasonably attributed to partial component dissolution or leaching, because CWPU0 lacks a crosslinked polymer network. In contrast, the castor oil-modified films (CWPU3.5–11.8) maintained their structural integrity and showed a net weight gain from solvent absorption rather than mass loss.

Particularly, CWPU3.5 showed the highest solvent uptake in THF. As this work represents an initial evaluation, this behavior is proposed to occur because THF has strong compatibility with the polyurethane matrix, and the low crosslinking density at 3.5 wt% CO presumably creates a loose network that allows a high volume of solvent to penetrate.

Regarding water resistance (Figure 6b), water absorption decreased progressively as the CO content increased, suggesting an improvement in stability. As the CO content increases from CWPU6.6 to CWPU11.8, the chemical resistance and water absorption show gradual improvements. This transition may be attributed to the potential 3D crosslinked network formed by the trifunctional castor oil, indicating that a denser network can help to reduce swelling [34,35].

Also, the surface wettability of the CWPU films was evaluated using static contact angle measurements. As shown in Figure 6c, all films exhibited contact angles below 90°, confirming an inherently hydrophilic nature. However, increasing the castor oil content led to a gradual rise in the contact angle from 43.85° to 49.28°, indicating a slight reduction in surface hydrophilicity. This trend may be due to the higher concentration of hydrophobic alkyl groups introduced by castor oil incorporation [19,36].

Overall, the water absorption values appear relatively high, approximately in the range of 60 to 75% when soaking for 48 h. Compared with other similar studies using CO with ionic agents—such as anionic dimethylol butyric acid (DMBA) or cationic N-methyl diethanolamine (MDEA)—the water absorption can reach <10% for 48 h with 90° of contact angles in the DMBA system, and <10% of water absorption for 20 days with <50° of contact angles in the MDEA system [32,37]. This comparison suggests that the nonionic A-130 puts this system at a relative disadvantage concerning water absorption.

#### 3.3.6. Weather Resistance (Jungle Test)

**Figure 7:**
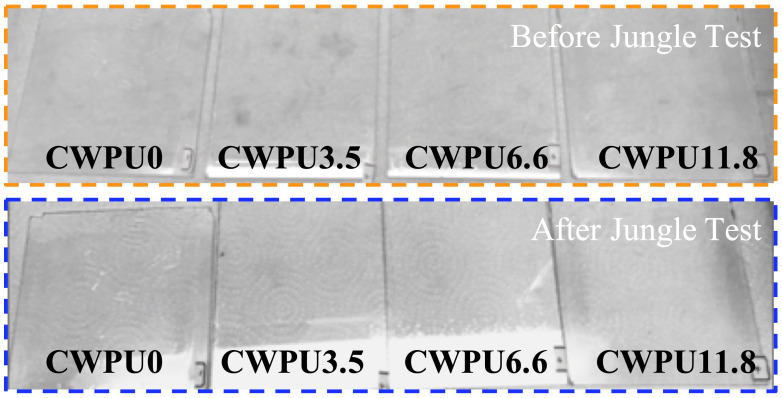
Visual appearance of CWPU coatings (CWPU0 to CWPU11.8) after accelerated ageing (jungle test) at 70 °C and 90% relative humidity for one week.

For a preliminary study on the environmental durability of the CWPU series, the samples were prepared via a doctor blade to obtain a coating thickness of approximately 7.5 μm. The test method followed ISO 1419 [38], commonly referred to as the “jungle test”. This accelerated aging procedure involved exposing samples (CWPU0 to CWPU11.8) to 70° and 90% relative humidity for one week, simulating long-term exposure to combined heat and moisture. As shown in Figure 7, the preliminary results show that no visible whitening, peeling, or surface degradation was observed in any of the tested samples [39].

## 4. Conclusions

In this study, a series of nonionic castor oil (CO)-modified waterborne polyurethanes (CWPUs) incorporating A-130 was successfully synthesized by a truly solvent-free process. The effects of CO content on the properties of the dispersions, mechanical performance, thermal stability of the dried films, and overall weather resistance as coatings were initially investigated. The results suggest that increasing the castor oil content led to positive effects on the overall performance. The prepared CWPU with a CO content of 11.8 wt% exhibited a dispersion particle size of 87.5 nm and a viscosity of 26.8 cP, maintaining excellent colloidal stability for at least 90 days under standard storage conditions. Furthermore, the maximum tensile strength reached up to 2.40 MPa with 570.37% of elongation, while the Td_5%_ and Td_10%_ reached 285.36 °C and 310.36 °C, respectively. However, the incorporation of A-130 led the overall CWPU series to exhibit a hydrophilic nature, with a maximum water contact angle of 49.3° and water absorption higher than 60% after 48 h. As for solvent resistance, the films showed less than 20% weight change in MEK and toluene, but showed greater swelling in THF over 24 h. For preliminary environmental durability, the CWPU coatings showed no visible whitening or peeling during a one-week standard jungle test. Due to the crosslinking and limited solubility of the CWPU series, standard GPC/SEC analysis was unfortunately not feasible. A thorough investigation involving DMA, gel fraction, or rheological testing will be pursued in our follow-up research to further differentiate the effects of crosslinking versus molecular weight changes. Overall, this work provides a new system of nonionic WPU combining CO and A-130 via an environmentally friendly synthesizing process without any organic solvents or volatile neutralizers, representing a sustainable strategy for the exploration of eco-friendly waterborne polyurethanes.

## Figures and Tables

**Table 1:** Ninety-day shelf life of CWPU dispersions.

	CWPU 0	CWPU 3.5	CWPU 6.6	CWPU 9.4	CWPU 11.8
0 day	V	V	V	V	V
10 days	V	V	V	V	V
20 days	V	V	V	V	V
30 days	V	V	V	V	V
40 days	V	V	V	V	V
50 days	V	V	V	V	V
60 days	V	V	V	V	V
70 days	V	V	V	V	V
80 days	V	V	V	V	V
90 days	V	V	V	V	V

**Table 2:** Crosslinking degree data of CWPU films.

Sample	V_p_ (cm^3^)	V_s_ (cm^3^)	V_2_	*n* (mol/cm^3^)
CWPU0	0.019	0.017	-	-
CWPU3.5	0.019	0.021	0.938	3.77 × 10^−2^
CWPU6.6	0.028	0.03	0.946	3.94 × 10^−2^
CWPU9.4	0.023	0.025	0.953	4.14 × 10^−2^
CWPU11.8	0.031	0.032	0.96	4.38 × 10^−2^

V_p_: Original sample volume, V_s_: Sample volume after soaking, V_2_: Ratio of original sample volume to swollen sample volume.

**Table 3:** Mechanical and thermal properties of the CWPU film series.

Sample	Tensile Strength (MPa)	Elongation at Break (%)	Td_5%_ (°C)	Td_10%_ (°C)	Td_max_ (°C)
CWPU0	1.45	1382.8	263.84	303.84	388.84
CWPU3.5	1.55	1152.7	268.90	303.90	395.95
CWPU6.6	1.64	1086.5	273.04	308.04	368.04
CWPU9.4	2.15	728.9	280.22	310.22	390.22
CWPU11.8	2.40	570.37	285.36	310.36	390.65

## Data Availability

The original contributions presented in this study are included in the article. Further inquiries can be directed to the corresponding author.

## Appendix A

**Table A1:** Equivalent ratio of CWPU series.

Sample Code	PTMG	CO	IPDI	A-130	EDA	Solid Content (%)	Bio-Based Content * (%)
CWPU0	1	0	2.52	0.4	0.56	35	0
CWPU3.5	0.9	0.07	2.46	0.4	0.55	35	3.5
CWPU6.6	0.8	0.13	2.4	0.4	0.53	35	6.6
CWPU9.4	0.7	0.2	2.34	0.4	0.52	35	9.4
CWPU11.8	0.6	0.27	2.28	0.4	0.5	35	11.8

* Bio-based contents (%) = (mass of castor oil)/(total mass of monomers) × 100.

**Table A2:** Results of NCO functional group titration of CWPU0.

Theoretical Value	0.0584			
Time	Blank Titration (V_0_)	Titration Endpoint (V_1_)	Sample Weight	Remaining NCO%
2.0 h	24.7	21.5	0.96	7.00%
2.5 h	24.7	21.1	1.1	6.87%
3.0 h	24.7	21.3	1.07	6.67%
3.5 h	24.7	21.5	1.05	6.40%
4.0 h	24.7	22.1	0.96	5.69%

**Table A3:** Results of NCO functional group titration of CWPU3.5.

Theoretical Value	0.0535			
Time	Blank Titration (V_0_)	Titration Endpoint (V_1_)	Sample Weight	Remaining NCO%
2.0 h	24.9	21.1	1.11	7.19%
2.5 h	24.9	21.3	1.11	6.81%
3.0 h	24.9	21.9	1.01	6.24%
3.5 h	24.9	21.9	1.06	5.94%
4.0 h	24.9	22.2	1.10	5.15%

**Table A4:** Results of NCO functional group titration of CWPU6.6.

Theoretical Value	0.0489			
Time	Blank Titration (V_0_)	Titration Endpoint (V_1_)	Sample Weight	Remaining NCO%
2.0 h	24.8	21.1	0.92	8.45%
2.5 h	24.8	21.2	1.04	7.27%
3.0 h	24.8	21.3	1.09	6.74%
3.5 h	24.8	22.1	1.05	5.40%
4.0 h	24.8	22.6	0.95	4.86%

**Table A5:** Results of NCO functional group titration of CWPU9.4.

Theoretical Value	0.0489			
Time	Blank Titration (V_0_)	Titration Endpoint (V_1_)	Sample Weight	Remaining NCO%
2.0 h	25.2	21.1	1.18	7.30%
2.5 h	25.2	22.0	1.09	6.17%
3.0 h	25.2	22.6	1.06	5.15%
3.5 h	25.2	22.8	1.11	4.54%
4.0 h	25.2	23.2	1.00	4.20%

**Table A6:** Results of NCO functional group titration of CWPU11.8.

Theoretical Value	0.0489			
Time	Blank Titration (V_0_)	Titration Endpoint (V_1_)	Sample Weight	Remaining NCO%
2.0 h	25.1	21.8	0.84	8.25%
2.5 h	25.1	22.3	0.84	7.09%
3.0 h	25.1	22.2	1	6.09%
3.5 h	25.1	23.1	0.88	4.77%
4.0 h	25.1	23.4	0.91	3.92%

**Table A7:** Viscosity and microparticle size of CWPU series dispersion.

Sample	Particle Size (nm)	PDI	Viscosity (cP)
CWPU 0	56.4	0.189	17.9
CWPU3.5	68.1	0.202	18.7
CWPU6.6	74.4	0.233	21.3
CWPU9.4	78.2	0.251	23.6
CWPU11.8	87.5	0.255	26.8
